# Use of whole genome sequencing to investigate an increase in *Neisseria gonorrhoeae* infection among women in urban areas of Australia

**DOI:** 10.1038/s41598-018-20015-x

**Published:** 2018-01-24

**Authors:** Cameron Buckley, Brian M. Forde, Ella Trembizki, Monica M. Lahra, Scott A. Beatson, David M. Whiley

**Affiliations:** 1 The University of Queensland, UQ Centre for Clinical Research, Herston, Queensland, 4029 Australia; 2 Australian Infectious Diseases Research Centre, The University of Queensland, Brisbane, Queensland 4072 Australia; 3 Australian Centre for Ecogenomics, The University of Queensland, Brisbane, Queensland 4072 Australia; 4 School of Chemistry and Molecular Biosciences, The University of Queensland, Brisbane, Queensland 4072 Australia; 5 WHO Collaborating Centre for STD, Microbiology Department, New South Wales Health Pathology East, Prince of Wales Hospital, Sydney, New South Wales 2031 Australia; 6 School of Medical Sciences, The University of New South Wales, Sydney, New South Wales 2052 Australia; 7 Microbiology Department, Pathology Queensland, Herston, Queensland 4029 Australia

## Abstract

Increasing rates of gonorrhoea have been observed among women within the Australian state of New South Wales. Here, we applied whole genome sequencing (WGS) to better understand the associated networks and transmission dynamics. Ninety-four isolates of a particular *N*. *gonorrhoeae* genotype (G122) associated with women (years 2012 to 2014) underwent phylogenetic analysis using core single nucleotide polymorphisms. WGS data revealed five main clusters, all of which were heterogeneous in terms of patient age and site of infection. The relatively high cervical/vaginal infections in each cluster was indicative of transmission in the general heterosexual population, noting that there is typically high rates of condom use for vaginal sex among local commercial sex workers. WGS also enabled the identification of groups of individuals belonging to tighter transmission chains within clusters, and hence may present a new tool for targeting public health interventions. The enhanced resolution of WGS provides a ready means of confirming suspected changes in *N*. *gonorrhoeae* epidemiology, but also enables key features to be identified or new questions to be raised regarding the composition of the associated sexual networks.

## Introduction

Notification rates for *Neisseria gonorrhoeae* infection in Australia have increased by 63% in the last 5 years, increasing from 61.9 per 100,000 in 2012 to 100.8 per 100,000 in 2016^1^. Gonorrhoea is primarily concentrated in two key populations in Australia: men who have sex with men (MSM) living in urban areas and Indigenous heterosexuals in regional and remote settings^2^. Until recently, there have been relatively low rates of gonorrhoea among heterosexuals living in urban areas of Australia^3,4^, but recent data show rates are now increasing among women in urban areas^1^. A retrospective study involving two sexual health clinics in urban Sydney, New South Wales (NSW), showed increasing rates among heterosexuals from 2008 to 2012^3^ that were associated with unprotected oral sex and commercial sex work (CSW). Our subsequent nationwide study of *N*. *gonorrhoeae* genotypes conducted using isolates from throughout Australia in 2012 revealed further evidence of gonorrhoea transmission among heterosexuals. This included identifying that the most common gonococcal strain was associated with heterosexuals and comprised 22% of all isolates in NSW, with a large proportion (54%) among women^5^. A further investigation^6^ showed this strain remained prevalent within NSW in 2014 and, although not remarked upon in the article, was prevalent among women. Combined, the above studies are highly suggestive of an emerging epidemic of gonorrhoea among urban heterosexuals in Australia. Confirming and also enhancing our understanding of these trends is important, particularly in light of the potential for increasing disease, including pelvic inflammatory disease among females.

A limitation of the genotyping protocol used in our previous studies was that whilst it targets 25 informative single nucleotide polymorphisms (SNPs) across the gonococcal genome, it lacks the added resolution offered by whole genome sequencing (WGS). WGS now has the ability to infer direct or indirect *N*. *gonorrhoeae* transmission links in the absence of traditional contact-tracing data^7^, as well as to reveal geographical and temporal spread and bridging across sexual networks of particular strains^8^. As highlighted by Didelot and colleagues^9^, it also provides enhanced resolution to more confidently assess likely transmission patterns among individuals, as opposed to using conventional typing tools which may lack appropriate discrimination. Here we aimed to utilise WGS to better understand transmission dynamics associated with an emerging strain of *N*. *gonorrhoeae* identified among women in urban areas of NSW, Australia. In doing so we aimed to assess the added value of using WGS over our conventional genotyping tool in understanding these trends. Here, we sequenced the genomes of all available isolates from women that belonged to the most common gonococcal genotype (G122; n = 94) identified in previous studies^5,6^. As an ancillary aim, we also assessed whether this genotype may be prevalent elsewhere by screening publically available *N*. *gonorrhoeae* isolate sequence data from previous studies in various geographical locations including Canada^10,11^, Brighton and surrounding areas in the United Kingdom^7^, mainland Europe^12^, Ireland^13^ and the United States^8^.

## Results

### Global prevalence and characteristics of genotype G122

*In silico* analysis of the NSW genomes identified that multilocus sequence type (MLST) 7359 was associated with genotype G122. Screening of the international collections for MLST 7359 identified only six isolates from Brighton (SRR3350090, SRR3343655, SRR3350214, SRR3350225 and SRR3343534, SRR3350146). Further *in silico* analysis of the six Brighton isolates revealed additional characteristics associated with G122, including the 345 A insertion in penicillin-binding protein 2 and wild-type resistance markers for the remaining genes that were screened (Supplementary Table 3). This coincides with G122 displaying a susceptible phenotype to all antibiotics^5^. The majority of NSW genomes (and all six Brighton genomes) were *N*. *gonorrhoeae* multi-antigen sequence type (NG-MAST) 4186. The remaining NSW isolates comprised four previously described NG-MAST profiles (NG-MAST 6759, n = 2; NG-MAST 6767, n = 2; NG-MAST 15344, n = 2 and NG-MAST 15348, n = 1) as well as one novel NG-MAST profile (NG-MAST 15609, n = 1). All NG-MAST profiles shared the same *tbpB* 241 allele but differed by their *porB* sequences which shared >99% nucleotide identity, indicating they all belonged to the same genogroup^14^.

To determine an appropriate outlying group to root the phylogeny comprising genotype G122, a core SNP phylogeny was generated from randomly selected isolates each representing a different MLST from the varying geographical locations (Supplementary Figure 1; Supplementary Table S5).

### Phylogenetic analysis of genotype G122

**Figure 1:**
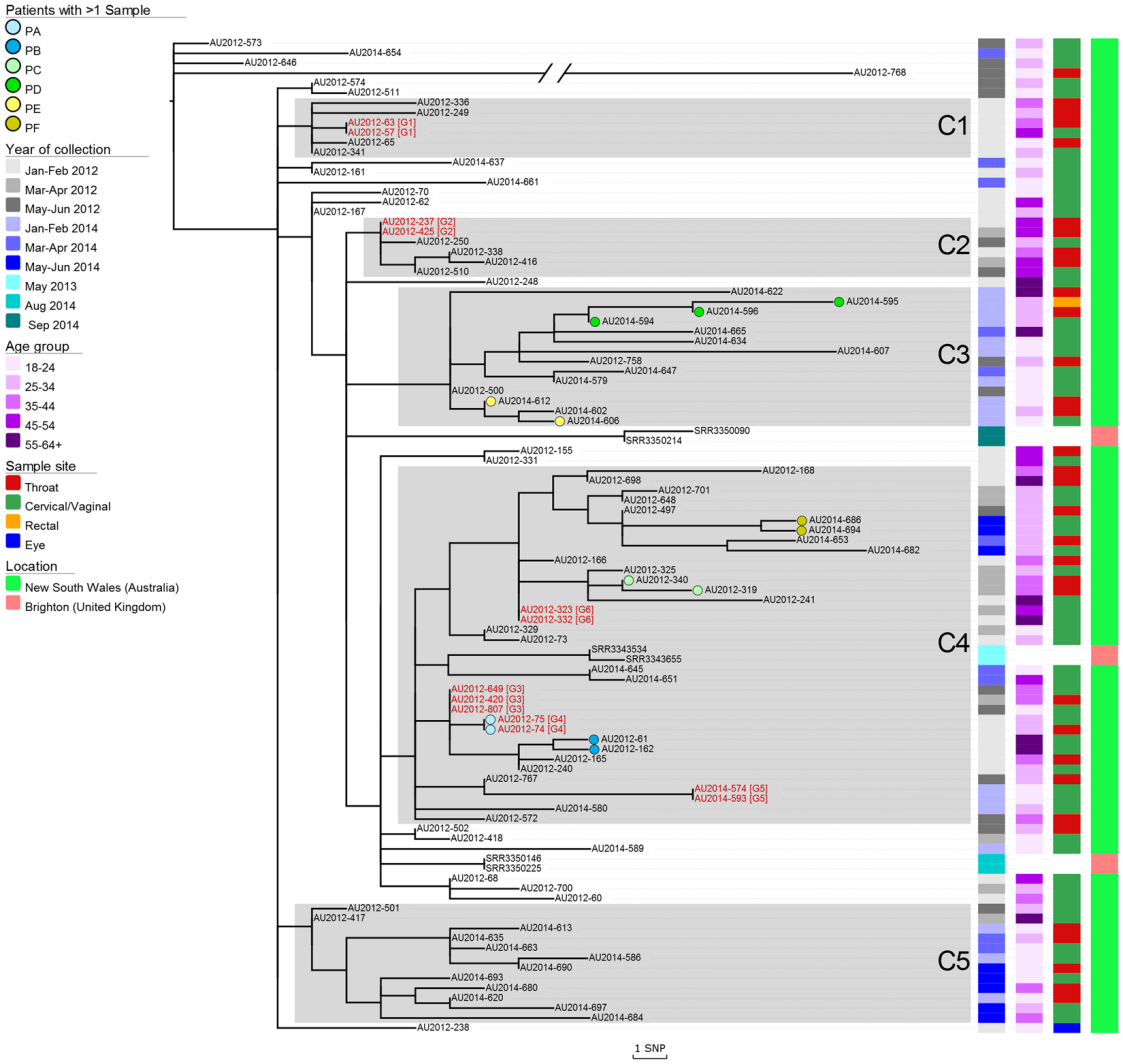
A rooted maximum likelihood phylogeny constructed with 473 core single nucleotide polymorphisms (SNPs). It comprises all 94 New South Wales (NSW) genomes and 6 Brighton genomes. A Brighton genome (SRR3360636) from the next closest sister clade was selected as the outlying group to root the phylogeny. Five larger clusters of isolates (C1-C5) have been labelled based on the phylogeny. Coloured circles represent isolates from the same patient (PA-PF; see key). Rectangles and their respective colour schemes correspond with date of collection, age group, sample site and location (see key). Both age group and sample site are unknown for the 6 Brighton genomes. The red isolate names with G1-G6 appended to the end, represent isolates that share identical core SNPs. Genome AU2012-768 has 33 core SNPs however its branch is truncated for easier visualisation purposes. The scale bar represents SNPs.

To investigate the genetic relationships within genotype G122 at the single nucleotide level, we determined a core SNP phylogeny of all 94 NSW genomes and the six highly similar Brighton genomes (Fig. 1). The core SNPs are based on the non-recombinant core genome derived from all samples included in the phylogeny. The phylogeny was rooted using an appropriate outlying group (Supplementary Figure 1). The majority of isolates belong to one of five clusters (C1 to C5). Each cluster, which comprised at least six isolates, contained one or more defining SNP or SNPs (being one or more SNPs shared by all isolates within a respective cluster, and not by isolates in other clusters) (Fig. 1). The maximum core SNP differences between genomes in each cluster, C1 to C5 respectively, were 6, 4, 19, 21 and 14 SNPs. Although the identified regions of recombination were highly similar for all isolates (Supplementary Figure 2), AU2012-768 comprised 33 unique core SNPs that were not shared by any other isolate in the phylogeny. Data regarding date of collection showed that three clusters (C3, C4 and C5) comprised isolates from both 2012 and 2014, whereas C1 and C2 comprised isolates from 2012. All clusters comprised varying patient age groups; age groups for C1 ranged from 18–24 to 45–54 years; C2 ranged from 25–34 to 45–54; and clusters C3 to C5 ranged from 18–24 to 55 years and over. The percentage of cervical/vaginal and throat samples respectively for each cluster were 33% and 67% for both C1 and C2, 57% and 36% for C3, 65% and 35% for C4 and 58% and 42% for C5 (summarised in Supplementary Table 1). No metadata was available for the six Brighton genomes except for date of collection. While two of these Brighton genomes were identified in cluster C4, the remaining four fell outside of assigned clusters, but still fell within the lineage.

### Diversity of G122 isolates within patients

Six individuals with more than one isolate were included in our study (Patients ‘PA’ to ‘PF’). The respective genomes from these six patients differed by less than eight core SNPs. Patient ‘PA’ had a throat and cervical swab collected on the same day, and was the only individual with more than one isolate that had indistinguishable core SNPs. Patients ‘PB’, ‘PC’ and ‘PD’ also had their respective isolates collected on the same day from different sample sites, but showed a difference of 2, 1 and 7 core SNPs, respectively. The remaining patients, PE and PF, each had different isolates collected between one and eleven days respectively and both differed by 2 core SNPs.

### Genetic links between patients

There were 6 instances where isolates had indistinguishable core SNPs and have been labelled as groups G1 to G6; these comprised either 3 isolates (G3) or 2 isolates each (G1, G2, G4, G5 and G6). Group G4 involved a single patient (patient ‘PA’ as described above) whereas the remaining five groups all involved different patients. The three isolates from group G3 were collected within 6 weeks of each other even though the collection dates provided in this study indicate a 3 month period (Supplementary Table 2). The isolates for groups G1, G2, G5 and G6 were all collected within one month of each other.

## Discussion

Overall the sequencing results show that isolates from the previously identified SNP-type are indeed closely related strains and, given they were all from women, provides further evidence of sustained transmission of this strain amongst heterosexuals. Moreover, the enhanced resolution provided by the phylogenetic analyses has provided new information regarding transmission of this strain within local sexual networks and abroad, and has potential implications for future use of real-time genomic surveillance for gonococcal infections.

This study included six patients (PA to PF) for which more than one isolate was collected. The observed SNP variation within each patient was found to be minimal, ranging from 0–7 core SNPs. This within-patient genomic stability is not surprising and is consistent with a previous study^7^. However, it provides an interesting context when considering the results for the five groups of patients (G1, G2, G3, G5 and G6; Fig. 1) all of which involved different individuals having identical core SNPs. Such results are suggestive of tight transmission networks, perhaps even common infection sources. Additional data would be needed to confirm the latter, given the links could be direct or indirect. Nevertheless, in the absence of these genomic data it would otherwise be very difficult to identify closely related transmission chains, as patients are often unwilling or unable to provide details regarding sexual partners^15^. Overall, the close relatedness among genomes within each cluster represent a high likelihood of transmission. Hence, these data highlight a potential role that WGS could have (assuming it could be achieved within a timely manner) in enhancing gonorrhoea management strategies, whereby the data is used to help pinpoint key groups of individuals for intervention. Ideally, this would involve using WGS in real-time to identify emerging clusters (including those that may involve newly identified resistant strains of public health importance), and using the data to target infected groups of individuals for intervention to limit further dissemination. The feasibility of this has already been highlighted by Todd *et al*.^16^ in an investigation of gonorrhoea in Newcastle, NSW in 2005. Albeit using a less discriminatory genotyping tool, Todd and colleagues were able quickly to identify the cluster and inform local medical practitioners. They subsequently ascertained risk of exposure and sexual contact information and identified an association with CSW. In response, there were renewed efforts to encourage sex workers to seek testing, which lead to successful control of the outbreak amongst heterosexuals.

The use of WGS, as outlined above, for identifying potential links between individuals in the context of sexually transmitted infections does of course raise some ethical concerns, including whether a patient’s privacy may be unduly comprised. In fact, some jurisdictions may ultimately prevent patients from being identified in WGS investigations of STIs, and only allow population-level trends to be ascertained using de-identified data (as applied in this research study). A counter-argument to these concerns is the fact that WGS cannot be used on its own to identify direct transmission events and that, even where isolates from two patients may share identical genomes, there could in fact be several different people in the transmission chain between the two patients. From a public health intervention perspective, there is probably little value in knowing whether there has been direct or indirect transmission. The imperative being to ensure as many individuals from a transmission chain are tested and treated, rather than identifying where in the chain individuals may sit.

The data also provided evidence that unprotected fellatio amongst CSW is not the sole driver perpetuating this G122 genotype in the population. Previous reports have shown high rates of condom use for vaginal sex, but lower rates for both oral sex and non-paying partners among CSW^17,18^. Hence, the fact that all clusters comprised a more even distribution of both pharyngeal and vaginal infections is inconsistent with known CSW practices, and more likely reflects transmission in the broader heterosexual population. These trends clearly warrant further investigation, and it may also be prudent to investigate whether rates of condom use for vaginal sex among CSW have changed. Nevertheless, being able to tease out the occurrence of genital and extra-genital infections within different clusters of patients is something that is not currently possible using conventional tools, and once again highlights the added value of WGS data in identifying new trends and new questions regarding sexual behaviour.

Of further interest in this study was the number of females in older age groups, with 21% of patients being 45 years or older. While there was some limited evidence of transmission within older ages (for example, cluster C2 predominately comprised individuals 45 years or older), the overall phylogeny suggested that gonorrhoea was not being sustained within distinct networks of older (≥45 years) individuals. Rather, the observed networks comprised a broad range of age groups and suggested that older individuals were acquiring infections that were otherwise being sustained in younger, more sexually active age groups. Currently, Australian federal policies promote sexual health to younger individuals and those of reproductive age as they are considered to be the most at risk for further complications^19^. Given our genomic data indicate networks of individuals comprising of varying ages, education on STI prevention may be more suited towards a broader range of age groups, including older ages and especially those engaging in new sexual relationships^20^.

Comparison with publically available genomic sequence data showed that genotype G122 was present in Brighton, United Kingdom, comprising just 0.4% of the Brighton sample bank, but was not present elsewhere. While these data suggest that the increases in the G122 genotype may be a purely Australian phenomenon, it needs to be recognised that currently-available *N*. *gonorrhoeae* genomic sequence data are somewhat limited. For example, genomic studies in Canada and the United States have focused on sequencing isolates of particular antimicrobial susceptibility profiles including cephalosporin and azithromycin resistance^8,10,11^. This selection would therefore exclude genotype G122 noting that G122 is sensitive to all tested antibiotics. Furthermore, the United States sample bank was acquired through the Gonococcal Isolate Surveillance Project which collects specimens from only men^21^, and therefore may be more representative of strains circulating in MSM rather than heterosexual networks. Likewise, even though the Brighton study did not specifically exclude isolates on the basis of antibiotic susceptibility or patient gender, it still comprised >90% men, and hence likely represents a predominately MSM-based population. Based on these selection biases and the susceptibility profile of G122, its absence from these samples banks was not surprising. Although determining the dissemination of key resistant strains is critical, being able to identify common circulating strains can also provide enhanced surveillance information^5^. A key example of this was identifying the six Brighton genomes associated with genotype G122. Their placement within the phylogeny may suggest transmission from NSW to Brighton, highlighting the associated risk for sexually active international travellers and the potential dissemination of strains. We note that MLST 7359 has been previously documented in Japan^22^ at relatively high prevalence (19.2% of assessed isolates), suggesting that G122 may indeed have some enhanced ability to transmit. Unfortunately, no sequencing data is available to further investigate these strains.

Currently, little is known as to why some *N*. *gonorrhoeae* strains may be more successful in a given population than others, but the advent of WGS now provides the potential to provide some insight into this. The fact that the G122 genotype lacked almost all commonly reported *N*. *gonorrhoeae* AMR mechanisms is consistent with the theory that bacteria may be more transmissible if not burdened by a plethora of AMR mechanisms, however for *N*. *gonorrhoeae* current thinking is that these AMR mutations do not necessarily confer a fitness disadvantage, as fitness can be restored via compensatory mutations^23^. Therefore it is likely that other factors, possibly unrecognised virulence mechanisms, may be important in sustaining G122 and other similar strains in the population.

There were several limitations associated with this study, firstly that we did not have access to behavioural data; and secondly males were not assessed. However, the decision to exclude males was deliberate and, in the absence of behavioural data, was done so as to focus on heterosexuals and limit any potential signal from MSM; Thirdly we only included isolates from two six-month time periods that were two years apart.

In summary, our genome sequencing data confirm sustained transmission of a particular strain of *N*. *gonorrhoeae* amongst women in urban areas of Australia from 2012 and 2014. Moreover, the enhanced resolution of WGS provides a means of readily identifying key features (or even raising new questions) regarding the composition of the sexual networks that would not otherwise be apparent through routine surveillance activities or through the use of our conventional genotyping method; for our G122 genotype this included showing that the strain was present in several distinct clusters, all of which were heterogeneous in terms of patient age, and that combined with site of infection data was indicative of transmission in the general heterosexual population, less so with commercial sex work. The ability to identify and characterise these clusters, including groups of individuals belonging to tighter transmission chains within, also presents new opportunities for targeted public health intervention. Overall, these data provide evidence of the additional value that WGS could provide in *N*. *gonorrhoeae* outbreak investigations. Studies aimed at identifying virulence markers that may be important in maintaining this strain in the population are ongoing.

## Methods

### *N*. *gonorrhoeae* isolates from NSW

A collection of 94 *N*. *gonorrhoeae* isolates, identified as genotype G122 by iPLEX massarray genotyping, were obtained from 87 women in NSW, Australia, as previously described^5,6^. The samples comprised 31 vaginal swabs, 27 cervical swabs, 33 throat swabs, 1 rectal swab, 1 eye swab and 1 genital swab. Age groups for the patients were; 18–24 years (n = 29), 25–34 (n = 31), 35–44 (n = 14), 45–54 (n = 11), 55 and over (n = 9). The isolates were from NSW, Australia and were isolated in January to June of 2012 (n = 59) and January to June of 2014 (n = 35). The isolate metadata is summarised in Supplementary Table 2. It should be noted that different names were used to describe these strains in the previous studies; the year 2012 isolates were originally described as belonging to strain-type “S01” and genotype “G122” (Supplementary Table 3 in^5^). The year 2014 isolates were called “NSW-001”. For consistency in this study, all 94 isolates are referred to as being of genotype G122.

### *N*. *gonorrhoeae* isolate sequence data from international collections

To provide global context and screen for the MLST associated with genotype G122, *N*. *gonorrhoeae* datasets from several international studies were also assessed. These include strains from Brighton and surrounding areas within the United Kingdom (n = 1,842)^7^, mainland Europe (n = 76)^12^, Ireland (n = 14)^13^, the United States (n = 236)^8^ and Canada (n = 169; n = 236)^10,11^.

### DNA extraction and sequencing for NSW isolates

A single colony from each isolate was cultured on LB agar and incubated at 37 °C, enriched with 5% CO_2_ for approximately 18–24 hours. Genomic DNA was extracted from half of a 10 µl loop of culture growth using the Ultraclean Microbial DNA Isolate Kit (GeneWorks, Australia), using the ‘Alternative Lysis Method’ as per manufacturer’s instructions. Libraries were prepared using the Illumina Nextera XT protocol, with 125 bp paired-end reads generated using the Illumina HiSeq instrument (AGRF, Melbourne, Australia), with HiSeq. 2500 V4 chemistry. Raw sequence reads from the 94 samples were assessed in FastQC v0.11.4^24^ and hard trimmed to 100 bp using Nesoni v0.132^25^.

### Assembly

The 94 NSW genomes were *de novo* assembled using SPAdes v3.6.2^26^. Sequence read and assembly metrics are summarised in Supplementary Table 4. Sequence read data for all NSW isolates were submitted to the NCBI Short Read Archive under BioProject PRJNA392203 (https://www.ncbi.nlm.nih.gov/bioproject/392203).

Genomes of interest from the international collections underwent *de novo* assembly using MegaHit v1.1.2^27^.

### MLST and NG-MAST assignment

Both the MLST and NG-MAST for all genomes were determined *in silico* using Ariba^28^ and NGMASTER^29^, respectively. For strains where sequence data was not available, MLST was performed using mlst v2.1^30^. Sequences with different NG-MAST profiles were compared using BioEdit v7.0.9.0^31^. Novel NG-MAST profiles were uploaded to the NG-MAST database (http://www.ng-mast.net/).

### Mutations associated with antimicrobial resistance

All 94 NSW genomes and those associated with genotype G122 underwent annotation via Prokka v1.11^32^. A combination of the method outlined below and BIGSdb^33^ were used to assess loci associated with antimicrobial resistance (AMR) including; *gyrA*, *folP*, *mtrR*, *parC*, *parE*, *penA*, *ponA*, *porB*, *rpoB*, *rpsE*, *rspJ* and 23 S rRNA. Using BLAST + and reference sequences derived from characterised WHO-F and WHO-U strains^34^, loci were extracted from an annotated multi-fasta file using seqtk^35^. Sequences were aligned and inspected using Clustal Omega^36^.

### Phylogenetic analyses

Phylogenetic trees were constructed using a core SNP alignment following the removal of recombinant regions. Draft genomes were aligned using Parsnp v1.2^37^, one of which was randomly selected to act as a reference, generating a core genome alignment. Recombinant regions were predicted and removed using Gubbins v2.1.0^38^. Phylogenetic trees were generated with RAxML v8.2.9^39^ using a general-time reversible nucleotide substitution model with a GAMMA correction for site variation. Phylogenetic trees were visualised using FigTree v1.4.2 and Evolview v2^40,41^.

### Data availability

The NSW sequence read data is available in the NCBI Short Read Archive repository; https://www.ncbi.nlm.nih.gov/bioproject/392203.

### Ethics approval

The study was approved by the South Eastern Sydney Local Health District Human Research Ethics Committees (HREC; LNR/14/POWH/146) and The University of Queensland HREC (2015001699), whereby a waiver of informed consent was included. All experiments were performed in accordance with relevant guidelines and regulations.

## Electronic supplementary material

Supplementary Information
