# Supplementary material for: Use of whole genome sequencing to investigate an increase in Neisseria gonorrhoeae infection among women in urban areas of Australia
Source: Sci Rep. 2018 Jan 24;8:1503. doi: 10.1038/s41598-018-20015-x (PMC5784116; doi:10.1038/s41598-018-20015-x)
Supplement: Supplementary file 1 — Supplementary Information [file 41598_2018_20015_MOESM1_ESM.pdf]

Use of whole genome sequencing to investigate an increase in *Neisseria gonorrhoeae* infection among women in urban areas of Australia.

Cameron Buckley[1], Brian M. Forde[2,3,4], Ella Trembizki[1], Monica M. Lahra[5,6], Scott A. Beatson[2,3,4] and David M. Whiley[1,2,7*].

[1] The University of Queensland, UQ Centre for Clinical Research, Herston, Queensland 4029, Australia.

[2] Australian Infectious Diseases Research Centre, The University of Queensland, Brisbane, Queensland 4072, Australia.

[3] Australian Centre for Ecogenomics, The University of Queensland, Brisbane, Queensland 4072, Australia.

[4] School of Chemistry and Molecular Biosciences, The University of Queensland, Brisbane, Queensland, 4072 Australia.

[5] WHO Collaborating Centre for STD, Microbiology Department, New South Wales Health Pathology East, Prince of Wales Hospital, Sydney, New South Wales 2031, Australia.

[6] School of Medical Sciences, The University of New South Wales, Sydney, New South Wales 2052, Australia.

[7] Pathology Queensland, Microbiology Department, Herston, Queensland 4029, Australia.

* Corresponding Author: David. M. Whiley. The University of Queensland, UQ Centre for Clinical Research, Herston, Queensland 4029, Australia. (d.whiley@uq.edu.au)

Table S1. Phylogenetic clusters and their associated metadata

Cluster	SNP range in each cluster (min-max)*	Isolate year of collection	Age groups	Cervical/vaginal sample %	Throat sample %
C1	0-6	2012 only	18-24 to 45-54	33%	67%
C2	0-4	2012 only	25-34 to 45-54	33%	67%
C3	1-19	2012 and 2014	18-24 to 55 and over	57%	36%
C4	0-21	2012 and 2014	18-25 to 55 and over	65%	35%
C5	1-14	2012 and 2014	18-25 to 55 and over	58%	42%

*SNP, single nucleotide polymorphism.

Table S2. New South Wales isolate metadata, genotyping and phylogenetic information

Strain ID	Specimen type	Age grouping	Collection (Month/Year)	Patient with >1 sample	MLST*	NG-MAST*	POR	TBPB	Cluster assigned in phylogeny	Strains sharing identical core SNPs*
AU2012-573	cervical/vaginal	25-34	05/2012		7359	4186	2569	241	Not assigned	
AU2014-654	cervical/vaginal	18-24	04/2014		7359	4186	2569	241	Not assigned	
AU2012-646	cervical/vaginal	25-34	05/2012		7359	4186	2569	241	Not assigned	
AU2012-768	throat	18-24	06/2012		7359	15344	8931	241	Not assigned	
AU2012-574	cervical/vaginal	25-34	06/2012		7359	4186	2569	241	Not assigned	
AU2012-511	cervical/vaginal	18-24	06/2012		7359	4186	2569	241	Not assigned	
AU2012-336	throat	35-44	02/2012		7359	4186	2569	241	C1	
AU2012-249	throat	25-34	02/2012		7359	4186	2569	241	C1	
AU2012-63	throat	35-44	01/2012		7359	4186	2569	241	C1	G1
AU2012-57	cervical/vaginal	45-54	01/2012		7359	4186	2569	241	C1	G1
AU2012-65	throat	18-24	01/2012		7359	4186	2569	241	C1	
AU2012-341	cervical/vaginal	25-34	02/2012		7359	4186	2569	241	C1	
AU2014-637	cervical/vaginal	18-24	03/2014		7359	4186	2569	241	Not assigned	
AU2012-161	cervical/vaginal	25-34	01/2012		7359	4186	2569	241	Not assigned	
AU2014-661	cervical/vaginal	18-24	04/2014		7359	4186	2569	241	Not assigned	
AU2012-70	cervical/vaginal	18-24	01/2012		7359	4186	2569	241	Not assigned	
AU2012-62	cervical/vaginal	45-54	01/2012		7359	4186	2569	241	Not assigned	
AU2012-167	cervical/vaginal	25-34	01/2012		7359	4186	2569	241	Not assigned	
AU2012-237	throat	45-54	02/2012		7359	4186	2569	241	C2	G2
AU2012-425	throat	45-54	03/2012		7359	15348	8935	241	C2	G2
AU2012-250	cervical/vaginal	25-34	05/2012		7359	6767	4059	241	C2	
AU2012-338	throat	35-44	02/2012		7359	4186	2569	241	C2	
AU2012-416	throat	45-54	04/2012		7359	4186	2569	241	C2	
AU2012-510	cervical/vaginal	45-54	05/2012		7359	4186	2569	241	C2	

Strain ID	Specimen type	Age grouping	Collection (Month/Year)	Patient with >1 sample	MLST*	NG-MAST*	POR	TBPB	Cluster assigned in phylogeny	Strains sharing identical core SNPs*
AU2012-248	cervical/vaginal	55 and over	01/2012		7359	4186	2569	241	Not assigned	
AU2014-622	throat	55 and over	02/2014		7359	4186	2569	241	C3	
AU2014-595	rectal	25-34	01/2014	PD	7359	4186	2569	241	C3	
AU2014-596	throat	25-34	01/2014	PD	7359	4186	2569	241	C3	
AU2014-594	cervical/vaginal	25-34	01/2014	PD	7359	4186	2569	241	C3	
AU2014-665	cervical/vaginal	55 and over	04/2014		7359	4186	2569	241	C3	
AU2014-634	cervical/vaginal	18-24	02/2014		7359	4186	2569	241	C3	
AU2014-607	cervical/vaginal	18-24	01/2014		7359	4186	2569	241	C3	
AU2012-758	throat	25-34	06/2012		7359	4186	2569	241	C3	
AU2014-647	cervical/vaginal	18-24	03/2014		7359	4186	2569	241	C3	
AU2014-579	cervical/vaginal	18-24	01/2014		7359	4186	2569	241	C3	
AU2012-500	cervical/vaginal	18-24	05/2012		7359	4186	2569	241	C3	
AU2014-612	throat	18-24	02/2014	PE	7359	4186	2569	241	C3	
AU2014-602	throat	25-34	01/2014		7359	4186	2569	241	C3	
AU2014-606	cervical/vaginal	18-24	01/2014	PE	7359	4186	2569	241	C3	
AU2012-155	throat	45-54	01/2012		7359	4186	2569	241	Not assigned	
AU2012-331	cervical/vaginal	45-54	02/2012		7359	4186	2569	241	Not assigned	
AU2012-168	throat	35-44	01/2012		7359	4186	2569	241	C4	
AU2012-698	throat	55 and over	02/2012		7359	4186	2569	241	C4	
AU2012-701	cervical/vaginal	25-34	03/2012		7359	4186	2569	241	C4	
AU2012-648	cervical/vaginal	25-34	03/2012		7359	4186	2569	241	C4	
AU2012-497	throat	25-34	05/2012		7359	4186	2569	241	C4	
AU2014-686	cervical/vaginal	25-34	06/2014	PF	7359	4186	2569	241	C4	

Strain ID	Specimen type	Age grouping	Collection (Month/Year)	Patient with >1 sample	MLST*	NG-MAST*	POR	TBPB	Cluster assigned in phylogeny	Strains sharing identical core SNPs*
AU2014-694	cervical/vaginal	25-34	06/2014	PF	7359	4186	2569	241	C4	
AU2014-653	throat	25-34	04/2014		7359	4186	2569	241	C4	
AU2014-682	cervical/vaginal	25-34	05/2014		7359	4186	2569	241	C4	
AU2012-166	throat	35-44	01/2012		7359	4186	2569	241	C4	
AU2012-325	cervical/vaginal	25-34	03/2012		7359	4186	2569	241	C4	
AU2012-340	throat	35-44	03/2012	PC	7359	4186	2569	241	C4	
AU2012-319	throat	35-44	03/2012	PC	7359	4186	2569	241	C4	
AU2012-241	cervical/vaginal	55 and over	02/2012		7359	4186	2569	241	C4	
AU2012-323	cervical/vaginal	45-54	03/2012		7359	4186	2569	241	C4	G6
AU2012-332	cervical/vaginal	55 and over	02/2012		7359	4186	2569	241	C4	G6
AU2012-329	cervical/vaginal	18-24	03/2012		7359	4186	2569	241	C4	
AU2012-73	cervical/vaginal	25-34	01/2012		7359	4186	2569	241	C4	
AU2014-645	cervical/vaginal	18-24	03/2014		7359	4186	2569	241	C4	
AU2014-651	cervical/vaginal	55 and over	03/2014		7359	4186	2569	241	C4	
AU2012-649	cervical/vaginal	35-44	05/2012		7359	4186	2569	241	C4	G3
AU2012-420	throat	35-44	03/2012		7359	4186	2569	241	C4	G3
AU2012-807	cervical/vaginal	18-24	05/2012		7359	4186	2569	241	C4	G3
AU2012-75	cervical/vaginal	25-34	01/2012	PA	7359	4186	2569	241	C4	G4
AU2012-74	throat	25-34	01/2012	PA	7359	4186	2569	241	C4	G4
AU2012-61	cervical/vaginal	55 and over	01/2012	PB	7359	4186	2569	241	C4	
AU2012-162	cervical/vaginal	55 and over	01/2012	PB	7359	6767	4059	241	C4	
AU2012-165	throat	35-44	01/2012		7359	4186	2569	241	C4	

Strain ID	Specimen type	Age grouping	Collection (Month/Year)	Patient with >1 sample	MLST*	NG-MAST*	POR	TBPB	Cluster assigned in phylogeny	Strains sharing identical core SNPs*
AU2012-240	cervical/vaginal	25-34	02/2012		7359	4186	2569	241	C4	
AU2012-767	throat	25-34	06/2012		7359	4186	2569	241	C4	
AU2014-574	cervical/vaginal	18-24	01/2014		7359	4186	2569	241	C4	G5
AU2014-593	cervical/vaginal	18-24	01/2014		7359	4186	2569	241	C4	G5
AU2014-580	cervical/vaginal	25-34	01/2014		7359	4186	2569	241	C4	
AU2012-572	throat	35-44	05/2012		7359	4186	2569	241	C4	
AU2012-502	throat	25-34	05/2012		7359	15609	9143	241	Not assigned	
AU2012-418	cervical/vaginal	18-24	03/2012		7359	4186	2569	241	Not assigned	
AU2014-589	cervical/vaginal	18-24	01/2014		7359	4186	2569	241	Not assigned	
AU2012-68	cervical/vaginal	45-54	02/2012		7359	4186	2569	241	Not assigned	
AU2012-700	cervical/vaginal	25-34	04/2012		7359	4186	2569	241	Not assigned	
AU2012-60	cervical/vaginal	35-44	02/2012		7359	4186	2569	241	Not assigned	
AU2012-501	cervical/vaginal	25-34	05/2012		7359	4186	2569	241	C5	
AU2012-417	cervical/vaginal	55 and over	03/2012		7359	4186	2569	241	C5	
AU2014-613	throat	18-24	02/2014		7359	4186	2569	241	C5	
AU2014-635	throat	25-34	03/2014		7359	6759	4057	241	C5	
AU2014-663	cervical/vaginal	18-24	04/2014		7359	15344	8931	241	C5	
AU2014-586	cervical/vaginal	18-24	01/2014		7359	4186	2569	241	C5	
AU2014-690	throat	18-24	06/2014		7359	6759	4057	241	C5	
AU2014-693	cervical/vaginal	18-24	06/2014		7359	4186	2569	241	C5	
AU2014-680	throat	35-44	05/2014		7359	4186	2569	241	C5	
AU2014-620	throat	18-24	02/2014		7359	4186	2569	241	C5	
AU2014-697	cervical/vaginal	25-34	06/2014		7359	4186	2569	241	C5	
AU2014-684	cervical/vaginal	35-44	05/2014		7359	4186	2569	241	C5	

Strain ID	Specimen type	Age grouping	Collection (Month/Year)	Patient with >1 sample	MLST*	NG-MAST*	POR	TBPB	Cluster assigned in phylogeny	Strains sharing identical core SNPs*
AU2012-238	eye	18-24	02/2012		7359	4186	2569	241	Not assigned	

*MLST, multilocus sequence type; NG-MAST, *Neisseria gonorrhoeae* multi-antigen sequence type; SNPs, single nucleotide polymorphisms.

Table S3. Resistance markers for all isolates comprising the G122 genotype.

Gene marker of Interest	Present/Absent or Wild-type/Mutant
PBP2 D345 ins	Present
porB1b G101	Wild-type
porB1b A102	Wild-type
gyrA S91	Wild-type
gyrA D95	Wild-type
parC D86	Wild-type
parC S87	Wild-type
parC S88	Wild-type
parE G410	Wild-type
rpoB H552	Wild-type
rpsJ v57	Wild-type
folP R228	Wild-type
ponA1 L421	Wild-type
mtrR G45	Wild-type
PBP2 A311	Wild-type
PBP2 I312	Wild-type
PBP2 V316	Wild-type
PBP2 T483	Wild-type
PBP2 G545	Wild-type
PBP2 A501	Wild-type
PBP2 G542	Wild-type
PBP2 P551	Wild-type
23S 2059*	Wild-type
23S 2611*	Wild-type

*Both 23S mutations were detected using BIGSdb (https://pubmlst.org/software/database/bigsdb/).

Table S4. Sequence read and assembly metrics for all New South Wales isolates

Strain ID	SRA Accession No.	Total bp	Ns (bp)	Total reads	Phred	Average quality	Depth	Contigs	Average contig size (bp)	Maximum contig size (bp)	N50 (bp)
AU2012-155	SRR5801862	2161415	187	3878998	33	34.1	211x	281	7691	207485	67601
AU2012-161	SRR5801863	2148888	172	4580264	33	34.3	249x	202	10638	208225	72540
AU2012-162	SRR5801864	2156075	172	3883220	33	34.5	212x	226	9540	207466	52268
AU2012-165	SRR5801865	2171196	329	4211752	33	34.6	228x	264	8224	207466	52955
AU2012-166	SRR5801851	2166498	550	3717494	33	33.8	202x	279	7765	207486	49447
AU2012-167	SRR5801866	2148757	174	4484230	33	34.6	243x	221	9722	207471	67601
AU2012-168	SRR5801867	2155756	174	4052462	33	34.6	220x	218	9888	207466	52403
AU2012-237	SRR5801879	2154101	174	3901672	33	34.5	212x	197	10934	211214	52438
AU2012-238	SRR5801881	2159696	172	4151712	33	34.6	225x	211	10235	207486	52430
AU2012-240	SRR5801880	2153010	180	3592010	33	34.5	195x	209	10301	207466	67556
AU2012-241	SRR5801878	2153907	7	3846260	33	34.5	208x	220	9790	207478	67556
AU2012-248	SRR5801868	2171941	173	4169658	33	34.7	226x	273	7955	207470	67556
AU2012-249	SRR5801877	2151488	173	3658062	33	34.6	199x	219	9824	207465	49045
AU2012-250	SRR5801808	2148699	179	5378114	33	34.2	291x	201	10690	211399	52438
AU2012-319	SRR5801893	2143904	172	3571752	33	34.3	194x	188	11403	207466	67601
AU2012-323	SRR5801892	2151119	174	3458586	33	34.6	188x	210	10243	207471	52430
AU2012-325	SRR5801876	2146381	173	3715372	33	34.5	202x	208	10319	207466	67644
AU2012-329	SRR5801895	2142204	174	3672470	33	34.2	200x	197	10874	207365	67556
AU2012-331	SRR5801883	2174483	174	3794450	33	34.8	206x	264	8236	207464	52430
AU2012-332	SRR5801871	2157811	174	4234652	33	34.5	230x	235	9182	207466	49411
AU2012-336	SRR5801870	2165094	174	4382356	33	34.7	237x	234	9252	207466	50074
AU2012-338	SRR5801882	2156764	174	3440720	33	34.5	187x	234	9216	207466	67556

Strain ID	SRA Accession No.	Total bp	Ns (bp)	Total reads	Phred	Average quality	Depth	Contigs	Average contig size (bp)	Maximum contig size (bp)	N50 (bp)
AU2012-340	SRR5801890	2147108	179	3765290	33	34.6	206x	224	9585	207486	72540
AU2012-341	SRR5801869	2153051	181	4111608	33	34.6	223x	215	10014	207466	52426
AU2012-416	SRR5801807	2151348	174	4364754	33	33.5	238x	208	10343	212034	67556
AU2012-417	SRR5801891	2149846	172	4677196	33	34.8	254x	222	9683	211762	52426
AU2012-418	SRR5801886	2150563	179	4586250	33	33.2	250x	222	9687	207466	57673
AU2012-420	SRR5801889	2157363	174	4306020	33	33.4	234x	222	9717	208226	53583
AU2012-425	SRR5801887	2152533	174	4169626	33	33.4	227x	213	10105	212048	53604
AU2012-497	SRR5801819	2144872	172	3507340	33	34.2	191x	230	9325	207466	67601
AU2012-500	SRR5801802	2149536	180	3878924	33	34.2	211x	216	9951	207467	67644
AU2012-501	SRR5801818	2147787	179	3557142	33	34.5	194x	215	9989	211831	67644
AU2012-502	SRR5801817	2149049	172	3620054	33	34.2	198x	234	9183	210651	67640
AU2012-510	SRR5801809	2150545	180	4013472	33	33.3	219x	189	11378	212409	72540
AU2012-511	SRR5801813	2154150	174	3622838	33	34	198x	218	9881	207486	67556
AU2012-57	SRR5801846	2154687	172	3164986	33	33.5	173x	208	10359	207486	66275
AU2012-572	SRR5801803	2154248	179	4113282	33	34.3	224x	219	9836	208224	67644
AU2012-573	SRR5801816	2149879	172	3816806	33	34.2	208x	207	10385	207365	67556
AU2012-574	SRR5801812	2150891	172	3995768	33	34.3	218x	229	9392	207486	67644
AU2012-60	SRR5801884	2145331	173	4998902	33	34.9	271x	195	11001	212044	67601
AU2012-61	SRR5801850	2156924	174	4197062	33	34.2	228x	235	9178	208226	52434
AU2012-62	SRR5801848	2172672	173	4219936	33	33.9	230x	270	8046	207466	49447
AU2012-63	SRR5801842	2160000	172	3680228	33	34.1	200x	246	8780	207466	49447
AU2012-646	SRR5801810	2151299	179	3828924	33	33	209x	206	10443	207466	67556
AU2012-648	SRR5801806	2147749	180	4271988	33	33.5	233x	225	9545	207478	67644

Strain ID	SRA Accession No.	Total bp	Ns (bp)	Total reads	Phred	Average quality	Depth	Contigs	Average contig size (bp)	Maximum contig size (bp)	N50 (bp)
AU2012-649	SRR5801811	2148807	182	4130786	33	33.7	225x	212	10135	207438	67556
AU2012-65	SRR5801845	2152675	173	3991296	33	33.8	218x	225	9567	207466	52399
AU2012-68	SRR5801885	2156012	174	3888914	33	34.7	212x	221	9755	207471	72540
AU2012-698	SRR5801894	2149342	172	3800734	33	34.4	207x	205	10484	207438	67556
AU2012-70	SRR5801849	2167537	173	3626668	33	33.9	198x	238	9107	207474	49447
AU2012-700	SRR5801805	2154061	174	4311810	33	33.2	235x	213	10112	212043	67556
AU2012-701	SRR5801888	2154257	174	4670058	33	33.5	254x	222	9703	208226	53604
AU2012-73	SRR5801847	2173798	171	3996638	33	33.8	218x	261	8328	207466	52977
AU2012-74	SRR5801843	2150880	174	3463202	33	33.8	189x	219	9821	207466	67603
AU2012-75	SRR5801844	2151104	183	3989426	33	34	217x	198	10864	207474	49393
AU2012-758	SRR5801820	2143260	132	3558356	33	34.3	194x	205	10454	207465	67556
AU2012-767	SRR5801814	2153686	174	3475670	33	34.5	190x	211	10207	207465	67556
AU2012-768	SRR5801815	2151064	173	3358624	33	34.2	183x	199	10809	207475	67601
AU2012-807	SRR5801804	2146673	174	4431142	33	33.3	242x	209	10271	207267	67601
AU2014-574	SRR5801821	2146883	174	5588486	33	35	301x	195	11009	207453	52420
AU2014-579	SRR5801823	2147032	172	3912014	33	34.5	214x	232	9254	207437	49069
AU2014-580	SRR5801822	2146436	172	3096932	33	34.1	169x	226	9497	207466	57525
AU2014-586	SRR5801825	2154795	174	3152682	33	34.3	172x	223	9662	207487	67556
AU2014-589	SRR5801824	2149095	173	3101244	33	34.2	169x	216	9949	207466	67556
AU2014-593	SRR5801827	2154287	183	3177564	33	34.5	173x	201	10717	207453	67549
AU2014-594	SRR5801826	2152505	172	3347582	33	34.3	183x	242	8894	207465	67556
AU2014-595	SRR5801829	2151008	173	3481212	33	34.3	190x	242	8888	207465	67556
AU2014-596	SRR5801828	2150788	173	3261190	33	34.3	178x	224	9601	207465	68045

Strain ID	SRA Accession No.	Total bp	Ns (bp)	Total reads	Phred	Average quality	Depth	Contigs	Average contig size (bp)	Maximum contig size (bp)	N50 (bp)
AU2014-602	SRR5801831	2149957	172	3227520	33	34.3	176x	221	9728	207466	67556
AU2014-606	SRR5801830	2152059	174	2708382	33	34.1	147x	197	10924	207466	69950
AU2014-607	SRR5801854	2152892	174	2706274	33	34.3	148x	212	10155	207466	67556
AU2014-612	SRR5801855	2149845	172	4123412	33	34.7	224x	201	10695	211764	49058
AU2014-613	SRR5801852	2155584	174	3277898	33	34.4	179x	224	9623	207486	72540
AU2014-620	SRR5801853	2151523	173	3270354	33	34.4	178x	214	10053	208662	67644
AU2014-622	SRR5801858	2152050	174	3163238	33	34.3	173x	202	10653	207466	68045
AU2014-634	SRR5801859	2150775	173	1806406	33	33.5	99x	213	10097	207437	52870
AU2014-635	SRR5801856	2149232	172	2519796	33	34.2	137x	196	10965	211506	67601
AU2014-637	SRR5801857	2152077	180	3032860	33	34.3	165x	206	10446	207487	72540
AU2014-645	SRR5801860	2149092	173	3139370	33	34.3	171x	216	9949	207497	67913
AU2014-647	SRR5801861	2147914	172	3233404	33	34.1	176x	210	10228	207437	67594
AU2014-651	SRR5801841	2144811	173	3050624	33	34.5	166x	203	10565	208230	66275
AU2014-653	SRR5801840	2144069	182	3005138	33	34.1	164x	201	10667	207466	52950
AU2014-654	SRR5801839	2144208	179	2763376	33	34.1	151x	217	9881	207439	66275
AU2014-661	SRR5801838	2142424	112	4697752	33	35.2	248x	196	10930	172608	47484
AU2014-663	SRR5801837	2149021	173	3710834	33	34.4	203x	221	9724	210620	67556
AU2014-665	SRR5801836	2146890	174	3691956	33	34.3	201x	210	10223	207467	67556
AU2014-680	SRR5801835	2147522	173	3878502	33	34.4	212x	211	10177	207497	52430
AU2014-682	SRR5801834	2147047	171	3623564	33	34.4	198x	211	10175	207467	67601
AU2014-684	SRR5801833	2145012	173	3165370	33	34.5	173x	199	10778	207465	67556
AU2014-686	SRR5801832	2155078	174	3573652	33	34.3	195x	213	10117	207471	52438
AU2014-690	SRR5801872	2151751	171	3954394	33	34.5	216x	222	9692	211488	67644

Strain ID	SRA Accession No.	Total bp	Ns (bp)	Total reads	Phred	Average quality	Depth	Contigs	Average contig size (bp)	Maximum contig size (bp)	N50 (bp)
AU2014-693	SRR5801873	2154484	179	3361948	33	34.2	184x	204	10561	207478	66275
AU2014-694	SRR5801874	2146778	173	3869124	33	34.6	211x	215	9985	207486	67594
AU2014-697	SRR5801875	2149156	172	3674642	33	34.3	201x	210	10234	211632	52430

Table S5. Randomly selected isolates representing each multilocus sequence type identified from international collections

MLST*	Lineage Colour	ID	Study
7359†	Black		De Silva et al. [1]
7826	Black	SRR3360636	De Silva et al. [1]
1594	Red	SRR3360958	De Silva et al. [1]
1892	Red	SRR3360668	De Silva et al. [1]
8135	Red	SRR3357253	De Silva et al. [1]
8394	Red	SRR3349949	De Silva et al. [1]
11675	Red	SRR1661263	Demczuk et al. [2]
11990	Red	SRR3360357	De Silva et al. [1]
12467	Red	SRR3343539	De Silva et al. [1]
12525	Red	SRR3360340	De Silva et al. [1]
12526	Red	SRR3360344	De Silva et al. [1]
12537	Red	SRR1661236	Demczuk et al. [2]
Novel-1	Red	SRR1661232	Demczuk et al. [2]
1596	Orange	SRR3350138	De Silva et al. [1]
11993	Orange	SRR3361170	De Silva et al. [1]
8122	Light green	SRR3361175	De Silva et al. [1]
8148	Light green	SRR3360836	De Silva et al. [1]
8163	Light green	SRR3349893	De Silva et al. [1]
12491	Light green	SRR3343580	De Silva et al. [1]
12507	Light green	SRR3360826	De Silva et al. [1]
12508	Light green	SRR3361326	De Silva et al. [1]
1595	Green	SRR3343571	De Silva et al. [1]
1906	Green	SRR3343590	De Silva et al. [1]
1918	Green	SRR3349722	De Silva et al. [1]
10316	Green	ERR191801	Grad et al. [3]
12527	Green	SRR1661169	Demczuk et al. [2]

MLST*	Lineage Colour	ID	Study
11191	Blue	SRR3361357	De Silva et al. [1]
11249	Blue	SRR3343519	De Silva et al. [1]
11516	Blue	SRR3361335	De Silva et al. [1]
1590	Cyan	SRR3361239	De Silva et al. [1]
6722	Cyan	ERR222899	Grad et al. [3]
8392	Cyan	SRR3360967	De Silva et al. [1]
10634	Cyan	SRR1661160	Demczuk et al. [2]
12506	Cyan	SRR3360647	De Silva et al. [1]
1584	Purple	SRR3360942	De Silva et al. [1]
1585	Purple	SRR3361323	De Silva et al. [1]
1599	Purple	SRR3349220	De Silva et al. [1]
1931	Purple	SRR3360630	De Silva et al. [1]
6960	Purple	SRR3360982	De Silva et al. [1]
8112	Purple	SRR3360831	De Silva et al. [1]
8114	Purple	SRR3360766	De Silva et al. [1]
8145	Purple	SRR3360979	De Silva et al. [1]
8154	Purple	ERR191779	Grad et al. [3]
9903	Purple	SRR3349206	De Silva et al. [1]
10317	Purple	SRR3349916	De Silva et al. [1]
11172	Purple	SRR3361300	De Silva et al. [1]
12492	Purple	SRR3343605	De Silva et al. [1]
12502	Purple	SRR3349967	De Silva et al. [1]
12503	Purple	SRR3350227	De Silva et al. [1]
12505	Purple	SRR3357184	De Silva et al. [1]
12536	Purple	SRR1661234	Demczuk et al. [2]
Novel-2	Purple	GCA_002081015.1	Mac Aogain et al. [4]
1927	Pink	SRR1661322	Demczuk et al. [5]
6714	Pink	SRR2736156	Demczuk et al. [5]

MLST*	Lineage Colour	ID	Study
10931	Pink	SRR3349845	De Silva et al. [1]
1580	Grey	SRR3349847	De Silva et al. [1]
1583	Grey	SRR3360646	De Silva et al. [1]
1587	Grey	SRR3350112	De Silva et al. [1]
1600	Grey	SRR3343639	De Silva et al. [1]
1893	Grey	SRR3343517	De Silva et al. [1]
1903	Grey	SRR3360848	De Silva et al. [1]
7360	Grey	SRR3349272	De Silva et al. [1]
7363	Grey	SRR3360833	De Silva et al. [1]
7365	Grey	SRR3343560	De Silva et al. [1]
7367	Grey	SRR3360921	De Silva et al. [1]
7371	Grey	GCA_001858695.1	Jacobsson et al. [6]
7822	Grey	SRR3343496	De Silva et al. [1]
7823	Grey	SRR3357139	De Silva et al. [1]
8110	Grey	ERR191735	Grad et al. [3]
8123	Grey	SRR3349919	De Silva et al. [1]
8126	Grey	SRR3360669	De Silva et al. [1]
8153	Grey	SRR3360956	De Silva et al. [1]
8156	Grey	SRR3349598	De Silva et al. [1]
8775	Grey	SRR3360713	De Silva et al. [1]
9362	Grey	SRR3357076	De Silva et al. [1]
9363	Grey	SRR3361108	De Silva et al. [1]
9365	Grey	SRR1661273	Demczuk et al. [2]
10312	Grey	ERR223625	Grad et al. [3]
10313	Grey	ERR191811	Grad et al. [3]
10314	Grey	ERR223658	Grad et al. [3]
10315	Grey	ERR222937	Grad et al. [3]
10622	Grey	SRR3360727	De Silva et al. [1]

MLST*	Lineage Colour	ID	Study
10886	Grey	SRR1661243	Demczuk et al. [5]
10891	Grey	SRR1661228	Demczuk et al. [2]
11231	Grey	SRR3343672	De Silva et al. [1]
11417	Grey	SRR3360745	De Silva et al. [1]
11420	Grey	SRR3349614	De Silva et al. [1]
11424	Grey	SRR2736217	Demczuk et al. [5]
11425	Grey	SRR2736154	Demczuk et al. [5]
11426	Grey	SRR1661207	Demczuk et al. [5]
11427	Grey	SRR2736205	Demczuk et al. [5]
11428	Grey	SRR3350168	De Silva et al. [1]
11463	Grey	SRR3357160	De Silva et al. [1]
11710	Grey	SRR3349622	De Silva et al. [1]
11864	Grey	SRR3349679	De Silva et al. [1]
11986	Grey	SRR3360906	De Silva et al. [1]
11999	Grey	SRR3361133	De Silva et al. [1]
12501	Grey	SRR3349716	De Silva et al. [1]
12504	Grey	SRR3357014	De Silva et al. [1]
12517	Grey	SRR3349981	De Silva et al. [1]
12521	Grey	SRR3350241	De Silva et al. [1]
12524	Grey	SRR1661163	Demczuk et al. [2]
12529	Grey	SRR3360924	De Silva et al. [1]
12530	Grey	SRR3361138	De Silva et al. [1]
12531	Grey	SRR1661179	Demczuk et al. [2]
12532	Grey	SRR1661194	Demczuk et al. [2]
12533	Grey	SRR3361307	De Silva et al. [1]
12542	Grey	SRR1661283	Demczuk et al. [2]
Novel-3	Grey	SRR1661170	Demczuk et al. [2]
Novel-4	Grey	SRR1661221	Demczuk et al. [2]

MLST*	Lineage Colour	ID	Study
Novel-5	Grey	SRR1661229	Demczuk et al. [2]
1579	Yellow	SRR3357137	De Silva et al. [1]
1582	Yellow	SRR3360912	De Silva et al. [1]
1588	Yellow	SRR3360625	De Silva et al. [1]
1597	Yellow	SRR3343586	De Silva et al. [1]
1601	Yellow	SRR3357272	De Silva et al. [1]
1901	Yellow	SRR3349848	De Silva et al. [1]
1902	Yellow	SRR3343505	De Silva et al. [1]
1904	Yellow	SRR3360917	De Silva et al. [1]
6959	Yellow	SRR2736142	Demczuk et al. [5]
7827	Yellow	SRR3350180	De Silva et al. [1]
8130	Yellow	SRR3350132	De Silva et al. [1]
8143	Yellow	SRR3350281	De Silva et al. [1]
8776	Yellow	SRR3350174	De Silva et al. [1]
8784	Yellow	SRR3360644	De Silva et al. [1]
10899	Yellow	SRR2736118	Demczuk et al. [5]
10932	Yellow	SRR3361315	De Silva et al. [1]
11194	Yellow	SRR3343615	De Silva et al. [1]
11421	Yellow	SRR3343669	De Silva et al. [1]
11956	Yellow	SRR3350271	De Silva et al. [1]
11967	Yellow	SRR3361223	De Silva et al. [1]
11979	Yellow	SRR3350164	De Silva et al. [1]
12500	Yellow	SRR3349197	De Silva et al. [1]
12519	Yellow	SRR3350175	De Silva et al. [1]

*MLST, multilocus sequence type.

†MLST 7359 comprised all 94 New South Wales genomes and six Brighton genomes; MLST Novel-1 to Novel-5 represent unassigned sequence types.

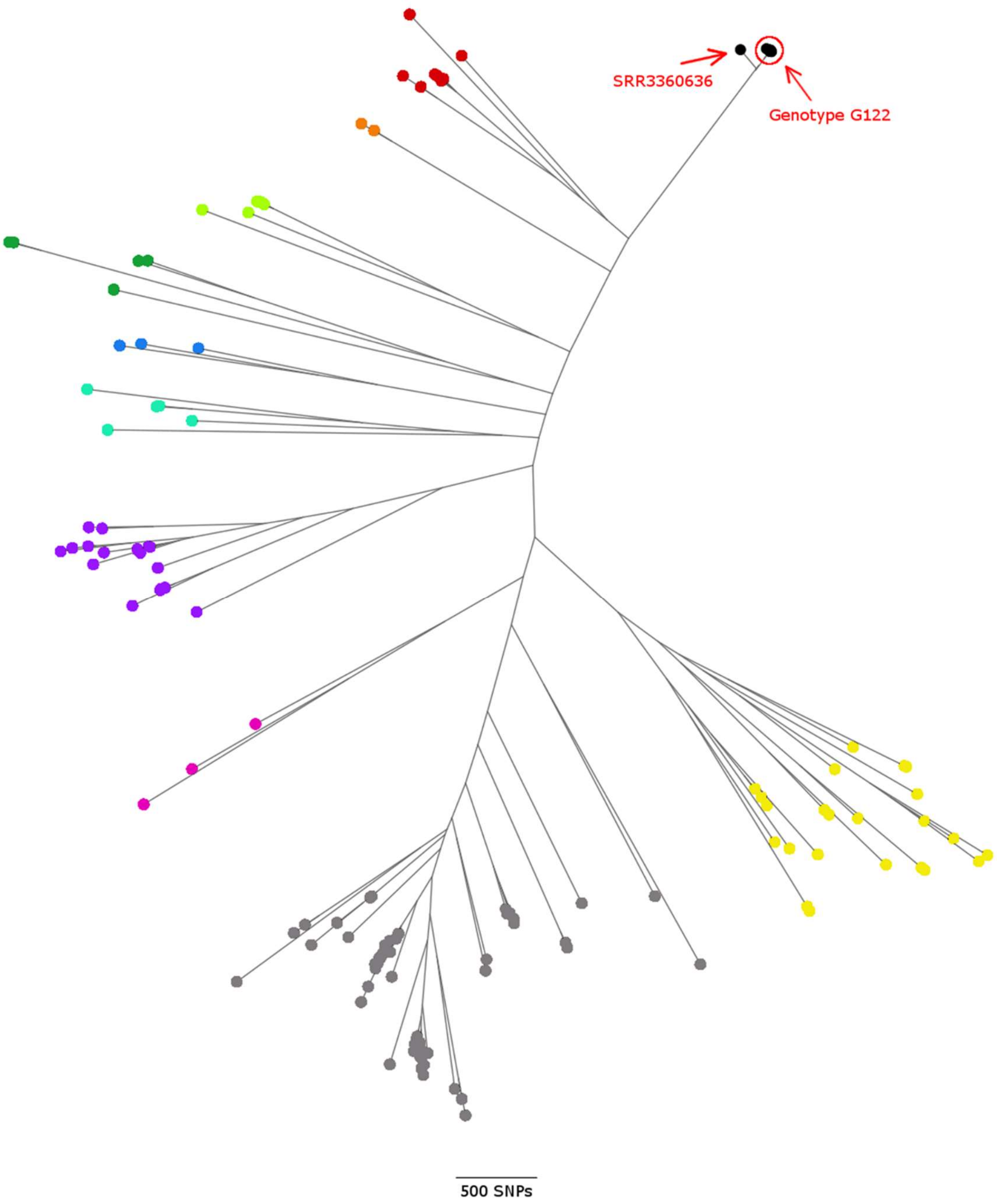

Figure S1. This unrooted maximum likelihood phylogeny is constructed from 16,805 core single nucleotide polymorphisms (SNPs). It comprises randomly selected isolates which are representative of distinct multilocus sequence types (n = 134) identified from varying *Neisseria gonorrhoeae* whole genome sequencing investigations conducted elsewhere (inlcuding Canada, Brighton and surrounding areas in the United Kingdom, mainland Europe, Ireland and the United States). See Supplementary Table S5 for each isolate and its corresponding sequence type and tip colour. All isolates representing genotype G122 form a tight cluster. SRR3360636 was the next closest related lineage and hence, was selected as an apprioriate outgroup to root the phylogeny depicted in Figure 1 of the main manuscript. The scale bar represents SNPs.

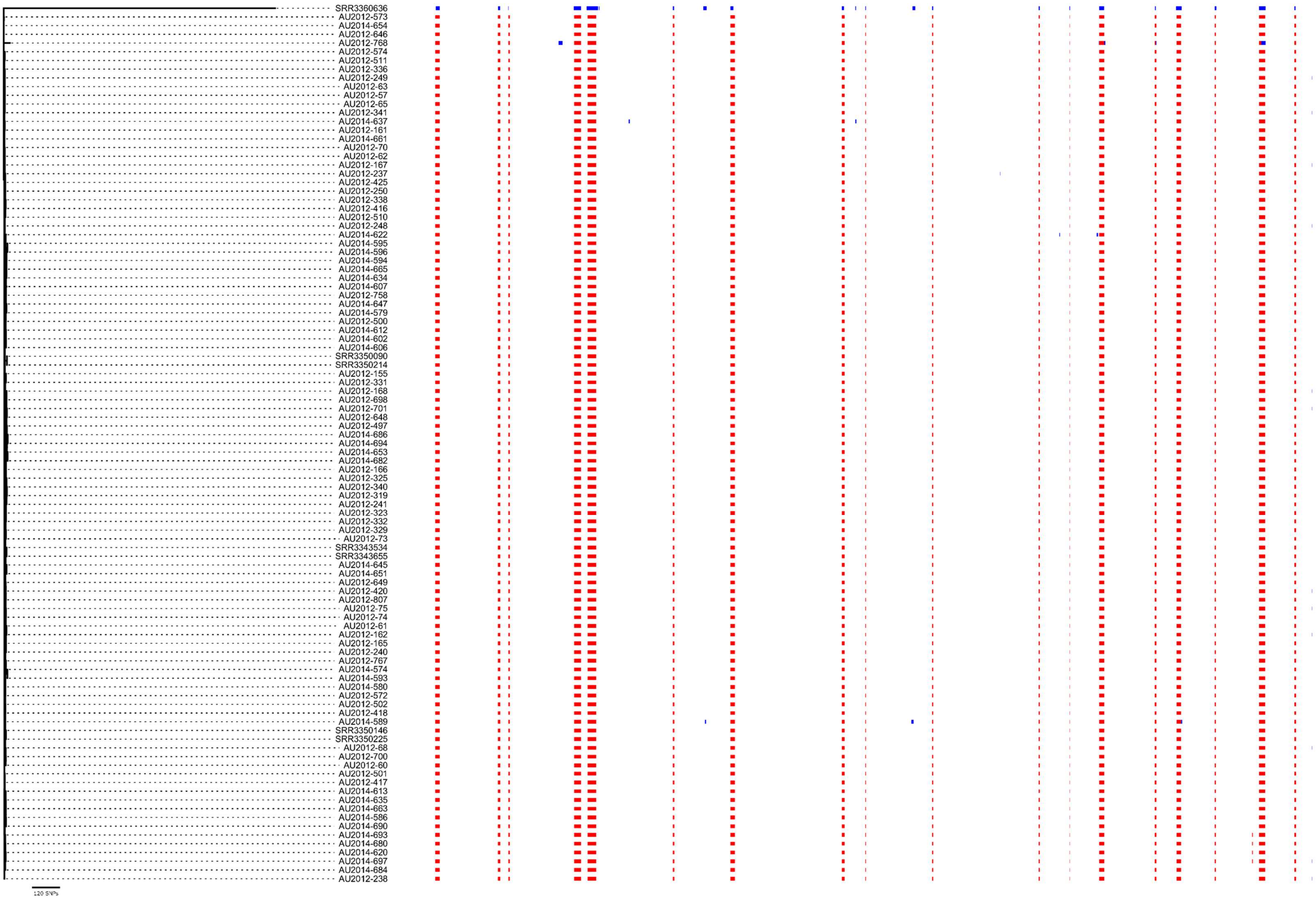
SRR3360636
AU2012-573
AU2014-654
AU2012-646
AU2012-768
AU2012-574
AU2012-511
AU2012-336
AU2012-249
AU2012-63
AU2012-57
AU2012-65
AU2012-341
AU2014-637
AU2012-161
AU2014-661
AU2012-70
AU2012-62
AU2012-167
AU2012-237
AU2012-425
AU2012-250
AU2012-338
AU2012-416
AU2012-510
AU2012-248
AU2014-622
AU2014-595
AU2014-596
AU2014-594
AU2014-665
AU2014-634
AU2014-607
AU2012-758
AU2014-647
AU2014-579
AU2012-500
AU2014-612
AU2014-602
AU2014-606
SRR3350090
SRR3350214
AU2012-155
AU2012-331
AU2012-168
AU2012-698
AU2012-701
AU2012-648
AU2012-497
AU2014-686
AU2014-694
AU2014-653
AU2014-682
AU2012-166
AU2012-325
AU2012-340
AU2012-319
AU2012-241
AU2012-323
AU2012-332
AU2012-329
AU2012-73
SRR3343534
SRR3343655
AU2014-645
AU2014-651
AU2012-649
AU2012-420
AU2012-807
AU2012-75
AU2012-74
AU2012-61
AU2012-162
AU2012-165
AU2012-240
AU2012-767
AU2014-574
AU2014-593
AU2014-580
AU2012-572
AU2012-502
AU2012-418
AU2014-589
SRR3350146
SRR3350225
AU2012-68
AU2012-700
AU2012-60
AU2012-501
AU2012-417
AU2014-613
AU2014-635
AU2014-663
AU2014-586
AU2014-690
AU2014-693
AU2014-680
AU2014-620
AU2014-697
AU2014-684
AU2012-238
120 SNPs

Figure S2. The predicted regions of recombination against the phylogeny produced through Gubbins. The red blocks are shared by all isolates in the phylogeny, while the blue blocks are unique to a particular isolate. With the exception of the outlying group (SRR3360636) at the top of the phylogeny, the remaining isolates (that represent genotype G122) share very similar predicted recombinant regions.

References

1 De Silva, D. *et al.* Whole-genome sequencing to determine transmission of Neisseria gonorrhoeae: an observational study. *Lancet Infect Dis* **16**, 1295-1303, doi:10.1016/S1473-3099(16)30157-8 (2016).

2 Demczuk, W. *et al.* Whole-genome phylogenomic heterogeneity of Neisseria gonorrhoeae isolates with decreased cephalosporin susceptibility collected in Canada between 1989 and 2013. *J Clin Microbiol* **53**, 191-200, doi:10.1128/JCM.02589-14 (2015).

3 Grad, Y. H. *et al.* Genomic epidemiology of Neisseria gonorrhoeae with reduced susceptibility to cefixime in the USA: a retrospective observational study. *Lancet Infect Dis* **14**, 220-226, doi:10.1016/S1473-3099(13)70693-5 (2014).

4 Mac Aogain, M. *et al.* Fourteen Draft Genome Sequences for the First Reported Cases of Azithromycin-Resistant Neisseria gonorrhoeae in Ireland. *Genome Announc* **5**, doi:10.1128/genomeA.00403-17 (2017).

5 Demczuk, W. *et al.* Genomic Epidemiology and Molecular Resistance Mechanisms of Azithromycin-Resistant Neisseria gonorrhoeae in Canada from 1997 to 2014. *J Clin Microbiol* **54**, 1304-1313, doi:10.1128/JCM.03195-15 (2016).

6 Jacobsson, S. *et al.* WGS analysis and molecular resistance mechanisms of azithromycin-resistant (MIC >2 mg/L) Neisseria gonorrhoeae isolates in Europe from 2009 to 2014. *J Antimicrob Chemother* **71**, 3109-3116, doi:10.1093/jac/dkw279 (2016).
